# LOVOT ROBOT AS COMPANIONS FOR OLDER ADULTS IN LONG-TERM CARE

**DOI:** 10.1093/geroni/igad104.3460

**Published:** 2023-12-21

**Authors:** Lillian Hung, Hiro Ito, Joey Wong

**Affiliations:** University of British Columbia, Vancouver, British Columbia, Canada; University of British Columbia, Vancouver, British Columbia, Canada; University of British Columbia, Vancouver, British Columbia, Canada

## Abstract

This exploratory, mixed-methods study explores how older adults living in Canadian Long-Term Care (LTC) homes experience and perceive LOVOT, an AI-driven social robot from Japan. It is an extended arm of a mixed-methods, three-country study conducted in Singapore, Hong Kong, and Canada. Our Canadian sample consists of 20 older adults and 40 interdisciplinary staff, and 10 leadership team members. The participants join four weekly sessions of interaction with LOVOT. In the quantitative portion of the study, questionnaires are administered before and after interaction with LOVOT to assess participants’ experiences of the LOVOT robot. The qualitative portion consists of individual conversational interviews with older adults and focus groups with the LTC staff and leadership. We use thematic analysis to guide our initial conceptual framework, and later use both Chi-square tests and content analysis for our quantitative and qualitative data. This study demonstrates (1) the experiences and perceptions of older adults and their family members regarding their interactions with the LOVOT robot, and (2) the LTC staff and leadership perceptions on having the LOVOT robot in LTC. The study offers insights into the potential role of social robots in LTC homes across eastern and western countries.

